# Novel Astrovirus Detected in Nasal Swabs of Sick Cattle, Mexico

**DOI:** 10.1111/irv.70226

**Published:** 2026-01-08

**Authors:** Judith U. Oguzie, Gustavo Hernandez‐Vidal, Gustavo Moreno‐Degollado, Lyudmyla V. Marushchak, Jessica Rodriguez, Gregory C. Gray

**Affiliations:** ^1^ Division of Infectious Diseases, Department of Internal Medicine University of Texas Medical Branch Galveston Texas USA; ^2^ Faculty of Veterinary Medicine Universidad Autónoma de Nuevo León Escobedo Nuevo León Mexico; ^3^ Department of Microbiology and Immunology University of Texas Medical Branch Galveston Texas USA; ^4^ Institute for Human Infections and Immunity University of Texas Medical Branch Galveston Texas USA

Dear Editor,

1

The *Astroviridae* family, historically associated with mild enteric and neurological symptoms, is now divided into two genera: *Mamastrovirus* (MAstV), which infects mammals, and *Avastrovirus* (AAstV), which infects avian species [1]. Three avastrovirus species are currently recognized to infect chickens, ducks, and turkeys. Nineteen mamastrovirus species (MAstV1–19) are currently recognized to infect diverse animal hosts, including cattle [1]. Four mamastroviruses (MAstV1, MAstV6, MAstV8, and MAstV9) have been found to also infect humans, further underscoring their zoonotic potential.

Bovine astrovirus (BoAstV) is an emerging group of unclassified mamastrovirus, namely, MAstV13, MAstV28–30, and MAstV33–35, of growing concern for cattle health and, within a One Health framework, may also have implications for human health [2]. Despite its apparent widespread distribution, the burden of BoAstV in cattle remains poorly defined. Limited surveillance and underdiagnosis likely obscure its contribution to enteric and respiratory disease, particularly in young calves where infection may drive morbidity, impair growth, and lead to economic losses. In addition, the epidemiology and genetic diversity of BoAstV in Latin America, and specifically in Mexico, remain largely uncharacterized.

Here we report, to our knowledge, the first detection and genomic characterization of BoAstV in cattle nasal swab specimens collected on Mexican farms. From February 2024 to May 2025, we conducted surveillance for novel coronavirus on Mexican cattle farms, where we collected nasal swabs from 40 cattle with respiratory signs. RNA was extracted from each nasal swab using the QIAamp Viral RNA Mini Kit on the QIAcube Connect automated system (QIAGEN Inc., Valencia, CA), and afterwards, we studied them with molecular assays for respiratory viruses. Among these samples, we selected 14 specimens for further study with metagenomic next‐generation sequencing based on molecular detection of influenza D virus or coronavirus via our pan‐coronavirus assay. Metagenomic sequencing libraries were prepared following a previously described workflow [3] and sequenced on either the Illumina NovaSeq X or Aviti platform. Sequencing data were analyzed on the CZ ID platform, and contigs with similarity to bovine astrovirus were identified and further characterized by BLAST analysis, phylogenetic reconstruction, and genotype assignment. Of the 14 specimens sequenced, we identified BoAstV contigs in three nasal swabs collected from cattle showing signs of respiratory disease, including fever and nasal discharge. Two of these specimens were also positive for influenza D virus, and one was positive for bovine coronavirus.

We aligned the three genomes (GenBank accession numbers PX692891–PX692893) with representative BoAstV genomes from NCBI using MAFFT v7.526 [4]. A maximum‐likelihood phylogenetic tree was reconstructed in IQ‐TREE using the GTR + F + G4 substitution model with 1000 bootstrap replicates to assess node support [5]. The resulting tree was annotated and visualized in FigTree v1.4.4 (https://tree.bio.ed.ac.uk/software/figtree/).

BLASTn analysis revealed that two genomes shared the highest nucleotide identity (98.24% and 98.54%) with ON552247, a novel astrovirus previously detected in bovine lung tissue from South Dakota. The third genome shared 95.74% nucleotide identity with KP264970, which was previously identified from a calf with respiratory disease, and 92.19% identity with ON552247. None of the sequences matched with the other astroviruses previously identified among the 19 mamastrovirus species. To our knowledge, these specimens represent the first BoAstV genomic sequences reported from cattle in Mexico and the only respiratory BoAstV genomic data from this region to date.

Consistent with their high nucleotide identity to the respiratory BoAstV strains ON552247 (21‐24401) and KP264970 (BSRI‐1), our three genomes clustered within the respiratory‐associated bovine astrovirus lineage (Figure 1), corresponding to Group 2 (G2) as defined by Zhu et al. [2].

**FIGURE 1:**
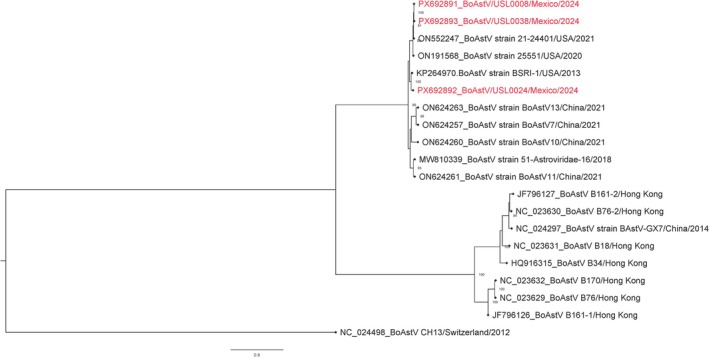
Maximum‐likelihood phylogeny of bovine astrovirus ORF1ab. A maximum‐likelihood (ML) tree was inferred from ORF1ab nucleotide sequences of bovine astroviruses to assess evolutionary relationships among study and reference strains. The tree was rooted to NC_024498. Branch lengths represent substitutions per site. Node support values are shown at key nodes. Cattle nasal swabs with novel astrovirus sequence from this study (PX692891–PX692893) are highlighted in red.

Although astroviruses are typically regarded as enteric pathogens, their roles in bovine respiratory and neurological disease are increasingly recognized. The detection of closely related BoAstV genomes in nasal swabs from cattle with respiratory illness in Mexico suggests that this lineage may contribute to the bovine respiratory disease complex. Furthermore, there are currently only a few respiratory BoAstV genomes available globally, and these respiratory strains appear genetically divergent from classical enteric astroviruses. Our findings emphasize the need for expanded genomic surveillance to understand the pathogenic potential and transmission dynamics of BoAstV, as well as its broader implications for cattle health and One Health.

## Author Contributions

**Judith U. Oguzie:** conceptualization, investigation, methodology, software, formal analysis, data curation, validation, writing – original draft. **Gustavo Hernandez‐Vidal:** investigation, writing – original draft, writing – review and editing, resources. **Gustavo Moreno‐Degollado:** investigation, writing – review and editing, resources. **Lyudmyla V. Marushchak:** investigation, methodology, writing – review and editing. **Jessica Rodriguez:** investigation, methodology, writing – review and editing. **Gregory C. Gray:** conceptualization, funding acquisition, writing – review and editing, investigation, writing – original draft, supervision.

## Funding

This project was supported in part by the Agriculture and Food Research Initiative Competitive Grant from the American Rescue Plan Act (award number 2023‐70432‐39558) through the USDA Animal and Plant Health Inspection Service (APHIS), USDA‐ARS Agreement 58‐3022‐4‐048, and by Professor Gregory C. Gray's startup funding from the University of Texas Medical Branch. The findings and conclusions in this report are those of the authors and should not be construed to represent any official USDA or US Government determination or policy.

## Data Availability

The data that support the findings of this study are openly available in GenBank at https://www.ncbi.nlm.nih.gov/genbank/about/, reference number PX692891‐PX692893.
